# Reparative dentin formation as a possible factor influencing the penetrability of dentin in human teeth with apical periodontitis: an ex vivo study

**DOI:** 10.1186/s12903-023-03105-9

**Published:** 2023-06-21

**Authors:** Yueyue Ren, Junsheng Zhang, Tiantian Meng, Wuli Li, Song Li

**Affiliations:** 1 College and Hospital of Stomatology, Key Lab of Oral Diseases Research of Anhui Province, Anhui Medical University, Hefei, 230032 China; 2 The First Affiliated Hospital of Anhui Medical University, Hefei, 230032 China; 3 Anhui Public Health Clinical Center, Hefei, 230032 China

**Keywords:** Dentine permeability, Infected root canal, Irrigation, Sodium hypochlorite, Reparative dentin

## Abstract

**Background:**

There is still a lack of knowledge regarding the permeability and configuration of infected root dentin. The aim of this ex vivo study was to compare the dentin penetrability of healthy teeth and necrotic teeth with apical periodontitis by evaluating the penetration of sodium hypochlorite (NaOCl) and to analyze the histopathological features of root dentin.

**Methods:**

Forty-eight molars were collected and divided into two groups. The clinical diagnosis for one group was pulp necrosis with apical periodontitis and the pulp and periapex were normal in the other group. Forty-eight straight roots were divided into two groups: infected and healthy. First, all root canals were stained with 2% methylene blue to visualize penetration after standard root canal instrumentation and irrigation. Transverse sections were obtained, and the dye penetration parameters were measured. The cross sections were processed to 20–30 μm and stained with hematoxylin and eosin for observation of the histopathological changes in the root dentin.

**Results:**

The maximum penetration depth, median penetration depth and penetration percentage of NaOCl solutions, in infected root canals were significantly lower than those in healthy root canals. The histopathological analysis showed that the frequency of reparative dentin formation in infected root canals was significantly greater than that in healthy root canals.

**Conclusions:**

The dentin penetrability of teeth with necrotic teeth and apical periodontitis was more superficial during root canal irrigation than that of healthy teeth. The histopathological changes in infected radicular dentin, namely the formation of reparative dentin, might be associated with the lower permeability of dentin tubules in human teeth with apical periodontitis.

## Background

The permeability of dentin, which is usually evaluated by the penetration depth of root canal irrigants into the dentin tubules, may affect the disinfection of the root canal system and the prognosis of the root canal treatment. Apical periodontitis is an infectious disease caused by microorganisms colonizing the root canal system, including dentinal tubules [1–3]. The microorganisms can invade dentinal tubules up to 1643 μm [4–9], making satisfactory disinfection of the root canal system challenging. Infected dentin is a potential source of microorganisms in persistent apical periodontitis [8–11]. Clinically, dentin tubule infection has been reported to occur in more than 75% of untreated teeth with primary apical periodontitis and 65% of teeth with enlarged canals [5]. Therefore, it is crucial to explore the factors that influence the penetration depth of root canal irrigants into infected dentin.

The penetration depth of irrigants into infected dentin depends on two aspects: namely the efficacy of different irrigation strategies and the permeability and configuration of the infected root dentin, including the number and diameter of the dentinal tubules. Over the past decade, endodontic research has focused on the irrigation strategies, such as the irrigation sequences [12–14] and activation techniques [15–17]. These studies that have investigated sodium hypochlorite (NaOCl) penetration into root dentin often use bovine incisors [14] or human mandibular premolars extracted for orthodontic reasons (age range of 14–20 years) [12, 15, 16]. The density and diameter of bovine dentin tubules are different from those of human dentin and may not reflect the true extent of dentinal infection in human teeth. Human dentin remains the preferred material for studying disinfection of dentinal tubules. Yet, most of the available teeth were removed from young people for orthodontic reasons, and these teeth typically contained a significant percentage of non-infected and more open dentinal tubules, which do not mimick clinical situations. Therefore, collecting human teeth extracted due to infected root canals as study subjects for irrigation experiments was more clinically representative.

The configuration of the infected dentin is another important factor that affects its permeability. However, little is known about the relationship between the permeability of infected radicular dentin and its configuration. Previous studies have shown that the permeability of healthy dentin is closely associated with physiological changes, and dentinal sclerosis is considered to be an important factor impeding the penetration of irrigants into the root canal system [18–21]. Recent studies showed that histological changes occurred in the radicular dentin of human teeth in the presence of deep caries, irreversible and reversible pulpitis, such as the formation of tertiary dentin on the canal walls [22–24]. It is well known that if left untreated, pulpitis usually progresses to pulp necrosis and periapical periodontitis. However, it remains unknown whether the above histological changes are also present in the infected root canals. In addition, it remains to be elucidated whether conformational changes affect the permeability of the infected radicular dentin.

In view of this, the primary aim of this ex vivo study was to compare the dentin permeability of healthy teeth and necrotic teeth with apical periodontitis by evaluating the penetration of NaOCl solutions. The secondary aim was to analyze histopathological features of radicular dentin in both groups to determine whether there was a link between dentin permeability and histopathological changes.

## Methods

### Specimen selection

This study was performed under the ethics approval (No: T2020008) of the College and Hospital of Stomatology of Anhui Medical University, and all methods were performed in accordance with the relevant guidelines and regulations. During sample collection, the pulpal/periapical status (radiolucent lesions/health) and patients age (< 30 years) were considered [21], while tooth morphology, root curvature, and etiology of the pulpal necrosis were evaluated. The teeth were confirmed via radiograph examination as having a sufficiently straight root canal. The exclusion criteria were a tooth having an apical foramen, root canal treatment, internal or external resorption, calcification, immature root apices, and/or a root canal curvature of greater than 10°. In accordance with these criteria and with considering the reduction of confounding bias, 48 multiple-rooted molar samples with relatively straight canals were collected and 48 straight roots were divided into two groups: **Group I** included infected root canals from subjects aged 19–30 years (teeth that responded negatively to pulp sensitivity test exhibited periapical radiolucency, and were extracted because of heavy destroyed), and **Group II** included healthy root canals from subjects aged 19–30 years (healthy teeth extracted for orthodontic reasons). Overall, 24 infected root canals and 24 healthy root canals were selected for the experiment. The teeth were submerged in 0.5% chloramine-T solution (Merck, Germany) for 2 days for decontamination and stored in distilled water until use.

### Experiment 1. Assessment of dentine permeability

In this ex vivo study, NaOCl solution penetration was used to assess the dentin penetrability of necrotic teeth with apical periodontitis with healthy teeth.

### Root canal instrumental preparation

Access cavities were prepared in healthy teeth with a diamond bur (MANI, Tochigi, Japan). To ensure the uniformity of access cavities, the teeth with infected root canals were restored with composite resin and extended coronally to create a simulated crown 6 mm in height, which is necessary for the activation methods.

A glide path was prepared with hand files from size 08, 0.02 taper to size 20, 0.02 taper (K-files, VDW, Munich, Germany). The working length was determined as the patency length minus 1 mm. The apex of each tooth was covered externally with light-cured flowable resin (3 M ESPE, St. Paul, MN, USA) to avoid overflow of the irrigating solutions and to simulate an anatomic condition in which the root apex is protected by periapical tissues. Root canals were instrumented in a crown-down manner with rotary files (VDW GmbH) up to apical size 40. Canals were irrigated with 3% NaOCl (SPEIKO, Munster, Germany) using a 31-G side-port needle (NaviTip; Ultradent, South Jordan, UT, USA), with a volume of 1 ml between files. NaOCl was removed with paper points, and teeth were stored in ultrapure water for less than 24 h before final irrigation.

### Irrigant activation protocol

The final irrigation solutions were 3% NaOCl, sterile saline, and 17% EDTA. The solutions were activated using an ultrasonic file in an ultrasonic generator (P5 NEWTRON, Servotome; Acteon, France) at power of 7 without water.

The protocol for final irrigation was as follows:

NaOCl (5 ml, 1 min).
2. Sterile saline water (5 ml, 1 min).
3. EDTA (5 ml, 1 min), activation for 30 s, resting phase of 30 s for two cycles.
4. Sterile saline water (5 ml, 1 min).
5. NaOCl (5 ml, 1 min), activation for 30 s, resting phase of 30 s, activation for 30 s for six cycles.

### Staining

The staining procedures were based on the model described by Galler et al. [16]. The outer surface of the teeth was covered with nail varnish to prevent external staining during the experiment, and the canal was filled with 2% methylene blue (BKMAM, Changde, China), which was activated for 1 min with PUI (a power of 7 and without water) at normal temperature to visualize the penetration depth of the irrigant. The canal was then dried with paper points and the teeth were stored dry for less than 72 h until further use.

### Sectioning and light microscopy

Teeth were embedded in acrylic Technovit 7200 resin (Heraeus Kulzer GmbH & Co. KG, Hanau, Germany), and transverse Sects. 100–150 μm thick were obtained from the center of the apical, middle, and coronal thirds of each tooth using an automatic grinder (EXAKT® cutting and grinding equipment: EXAKT® Apparatebau GmbH & Co., Norderstedt, Germany) under continuous water irrigation. All cross sections were examined with a light microscope (Zeiss Axiophot; Carl Zeiss, Oberkochen, Germany) connected to a digital camera (Zeiss Axiocam ERc 5s; Carl Zeiss) operated with AxioVision software (Carl Zeiss). For standardization of the images and to obtain subsequent reliable measurements, images were taken with the same objective at a fixed resolution and optimal focus. Images were acquired with a calibrated scale bar. The slices were analyzed with Image J software (National Institutes of Health, Bethesda, MD, USA) after calibration.

The penetration depths of methylene blue into the dentinal tubules were a surrogate measure of the penetration of the irrigants during activation. The effect of dye penetration was evaluated by measuring the median penetration depth (along 24 exactly spaced lines of an imaginary clock face. The penetration depth of methylene blue dye was determined visually by two examiners. The median penetration depth, maximum penetration depth, and dye penetration percentage (the percentage of the blue-stained portion of the root canal as part of the whole circumference) was calculated. All measurements were calculated and depicted for the coronal, middle and apical regions, and the median values and 25–75% quantiles of these values were also calculated. All sections were evaluated blindly. Additionally, to exclude errors during the analysis of the penetration depths, the same two examiners performed the task together. One examiner determined the start and end-points of each line segment, and the second examiner verified the correct procedure. The actual measurement of the line segments was determined using software.

### Experiment 2. Evaluation of histopathological changes in the root dentin [25]

All samples that assessed in Experiment 1, were used to analyze the histopathological features of radicular dentin in both groups. These cross-sections were ground to a 20- to 30-µm thickness using EXAKT grinding equipment and were then stained with haematoxylin and eosin to observe changes in the root dentin. Morphological changes in the root dentin were evaluated by independent, blinded, operators. The two blinded evaluators assessed the presence or absence of morphological changes in root dentin on each section according to the criteria for reparative dentin: sometimes the number of dentin tubules is significantly lower than in normal dentin, while the tubules are significantly curved, with some areas containing only a few tubules or sometimes osteodentin with no tubules and odontoblasts embedded in the interstitium leaving a gap in the place, much like bone tissue [18, 22–24]. When discrepancies occurred between operators, a consensus was reached by discussion. Thirty images were randomly selected after 4 weeks and submitted to a new assessment by the same evaluators in order to test for intra-observer agreement.

### Statistical analysis

All statistical analyses were performed using SPSS (v.23) software (IBM, Armonk, NY, USA). For experiment 1, independent samples t-tests or nonparametric tests were used with an α = 0.05 level of significance, and results are shown as median (1st quantile, 3rd quantile). For Experiment 2, data were evaluated with Pearson’s chi-square test to analyze the incidence of changes in the root dentin. Each third of the tooth was considered independent of the others.

## Results

### Assessment of dentine permeability

**Fig. 1:**
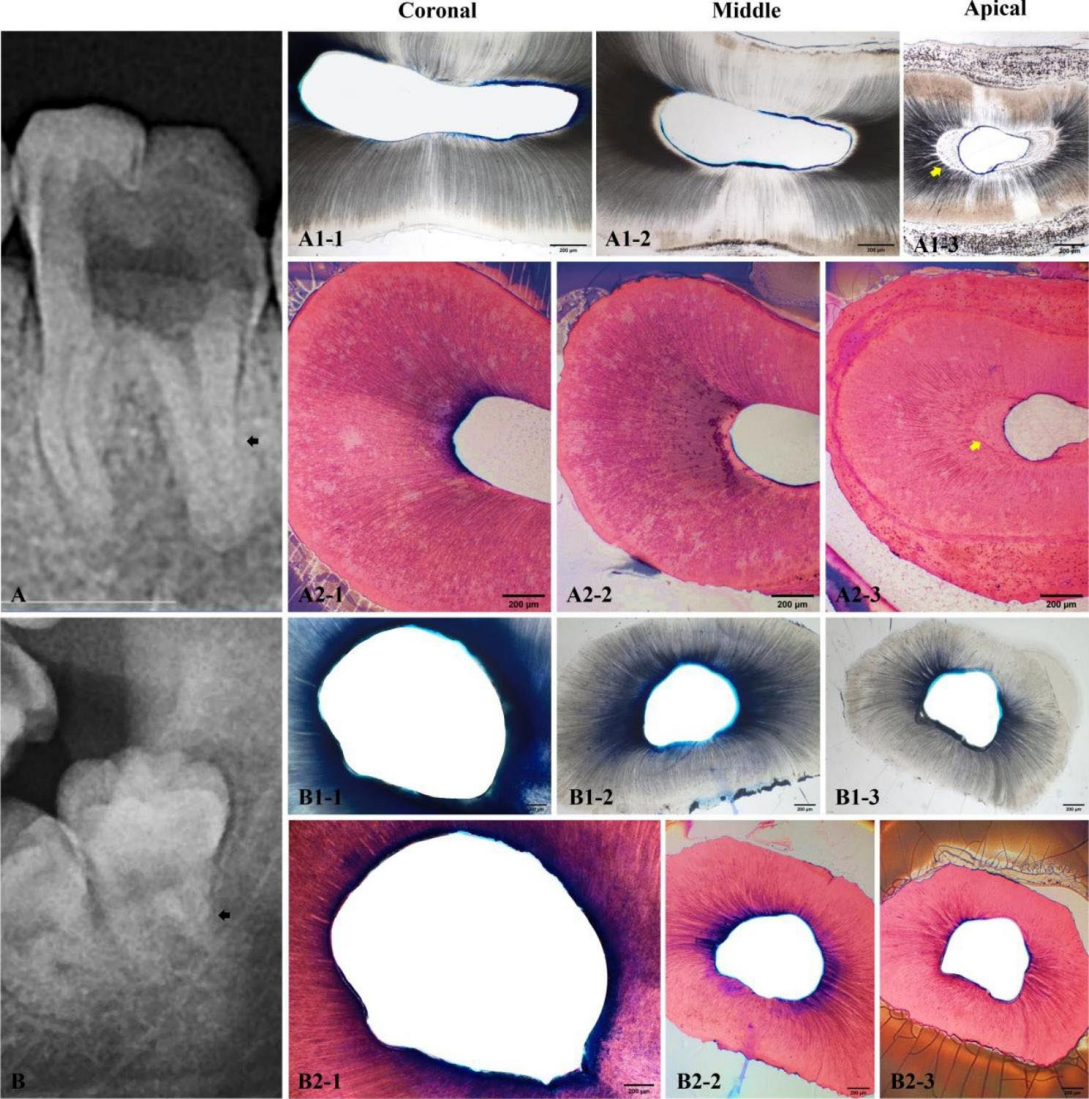
Representative images of dye penetration and hematoxylin–eosin (HE) staining, revealing that the dye penetration is more superficial in (A) the infected root canals than (B) root canals with normal healthy pulp. (A2) The reparative dentine, influences the penetrability of root dentine. **(A)** Left mandibular first molar of a 19-year-old woman with a destroyed crown and periapical lesions. **(A1-1/2/3)** Results of methylene blue staining in the distal root canal (black arrow in A). **(A2-1/2/3)** Results of HE staining of the same section. Yellow arrow indicates reparative dentine, formation in the apical section. **(B)** Healthy tooth 38 (left mandibular third molar) of a 24-year-old woman. **(B1-1/2/3)** Images of methylene blue staining of the distal root canal (black arrow in B). The dye is spreading evenly through the dentin tubules. **(B2-1/2/3)** HE staining of the same section, absent of reparative dentin

Based on the NaOCl solution penetration, the dentin penetrability of teeth with necrotic and apical periodontitis was lower than that of healthy teeth. The maximum penetration depth, median penetration depth, and penetration percentage in infected root canals were significantly lower than those in healthy root canals (*P* < 0.05). The results for maximum penetration depth, median penetration depth, and penetration percentage are summarized in Table 1. Figure 1 shows representative images of 2% methylene blue penetration into infected and healthy dentin (Fig. 1A 1**/2/3 and B-1 1/2/3**).

**Table 1:** Results of the maximum penetration depth, median penetration depth and penetration percentage

Root Canals	Median Penetration Depth (µm)			Maximum Penetration Depth (µm)			Penetration Percentage (%)		
	Coronal	Middle	Apical	Coronal	Middle	Apical	Coronal	Middle	Apical
Infected	22.1 (14.5–81.2)	24.8 (11.5–34.6)	9.0 (0.00–17.0)	248.3 (120.3–330.0)	202.8 (86.7-345.2)	71.8 (59.0-147.9)	84.9 (63.9–92.4)	88.6 (56.1–95.2)	60.8 (36.2–77.5)
Healthy	354.7 (175.7-524.9)	260.4 (129.1-403.4)	88.4 (16.5-257.5)	697.3 (442.2-916.4)	510.3 (350.6-725.5)	441.2 (299.8-571.1)	100.0 (97.4–100.0)	100.0 (91.0-100.0)	100.0 (89.1–100.0)
*P*	*P* < 0.001*	*P* < 0.001*	*P* < 0.001*	*P* < 0.001*	*P* < 0.001*	*P* < 0.001*	*P* < 0.001*	*P* < 0.001*	*P* < 0.001*

Results are presented as median (1st quantile, 3rd quantile). *The significance level is 0.05

### Histopathological changes in radicular dentin

**Fig. 2:**
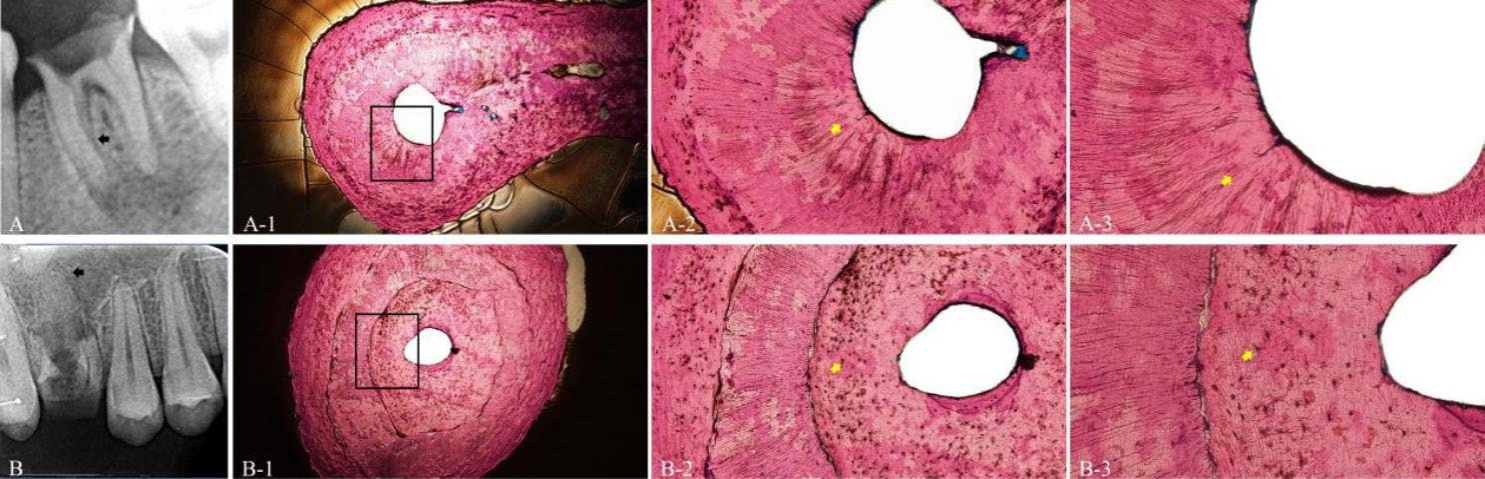
Various reparative dentin was deposited on the wall of the root canal, impeding dye penetration into the infected dentin. **(A)** Tooth 36 (left mandibular first molar) with periapical lesions in a 23-year-old man. **(A-1)** Apical section of mesial root canal (black arrow in A) (×40). **(A-2/3)** Magnifications of the area of the root canal wall indicated by the box in (A-1) (×100 and ×200), showing the reparative dentin with disordered and curved tubules clearly demarcated from normal dentin, resembling mineralized fibrous scar tissue (yellow arrow). **(B)** Tooth 16 (right maxillary first molar) with periapical lesions in a 21-year-old woman. **(B-1)** Apical section of palatal root canal of tooth B (black arrow in B) (×40). **(B-2/3)** Progressive magnifications of the area of the root canal wall indicated by the box in (B-1) (×100 and ×200), showing osteodentin, presenting traces of cells in its bone-like structure and no tubules in its matrix (yellow arrow)

Various degrees of reparative dentin formation were observed in both groups (Table 2). The frequency of reparative dentin formation in infected root canals was significantly greater than in healthy root canals. Some of the dentin resembled mineralized scar-like structure tissue with significantly disordered and curved tubules (Fig. 2A-1,2,3). . Others dentin presented as osteodentin (Fig. 2A2-1/2/3, Fig 2B-1,2,3), with traces of cells in its bone-like structure and no tubules. In the coronal, middle and apical regions of the infected root canals, the frequency of the reparative dentin formation was 85.00%、70.83% and 79.17%, respectively, which was greater than that of the healthy root canals (8.33%, 41.67%, and 50.00%, respectively; *P* < 0.05) (Fig. 2B2-1/2/3).

**Table 2:** Distribution of the reparative dentin in the infected and healthy root canals

Groups	Region	Number of sections containing reparative dentin/ Sum	Frequency (%)	*P*
Infected	Coronal	17/20^#^	85.00	*P* < 0.001*
	Middle	17/24	70.83	*P* = 0.042*
	Apical	19/24	79.17	*P* = 0.035*
Healthy	Coronal	2/24	8.33	
	Middle	10/24	41.67	
	Apical	12/24	50.00	

^#^There are four cases with decayed coronal section. *The significance level is 0.05

## Discussion

This study was a pilot investigation to compare the dentin permeability of healthy teeth and necrotic teeth with apical periodontitis, and to analyze histopathological features of radicular dentin in both groups.

Our results indicated that the dentin permeability of teeth with necrotic and apical periodontitis was more superficial than that of healthy teeth. Several previous studies have assessed the NaOCl penetration into human dentin tubules and showed that the median penetration depths by PUI amounted to > 500 μm for 3% NaOCl [12, 15, 16]. Consistent with the above studies, ultrasonic activation also resulted in significantly deeper irrigant penetration in healthy teeth, amounting to > 600 μm in this study. However, lower penetration depths occurred in teeth with apical periodontitis, the amounting only < 300 nm. To our knowledge, this study is the first to investigate NaOCl penetration in association with clinical conditions in which bacteria and their by-products have penetrated deeply into the dentinal tubules. These findings indicate that the dentin permeability of teeth with apical periodontitis is lower in clinical practice.

Our results showed that various reparative dentin was more frequently observed in the infected root canals than in healthy ones. The phenomenon of reparative dentin on the canal walls has been well documented, but it has never been the focus of attention in previous studies on irrigants [22–24]. Ricucci et al. examined the histological events that occurred in the radicular pulp of human teeth in the presence of deep caries, and irreversible, and reversible pulpitis, and they were the first to observe varying amounts of tertiary dentin on the canal walls [24]. The results showed that the histopathologic changes of dentine were observed in 28.6% of the intact mature teeth and 100% of all cases clinically diagnosed as irreversible and reversible pulpitis [23]. Consistent with Ricucci et al., our data showed that reparative dentin formation was more frequently present, to varying degrees, in infected canals (> 70%) than in healthy canals (< 50%). Some of the reparative dentin presented as osteodentin without tubules (Fig. 1A2-1/2/3, Fig. 2B1/2/3). Others presented with disordered and curved tubules (Fig. 2A1/2/3). As it is well known that most pulp necrosis and apical periodontitis develop from pulpitis, our results suggested that rootward diffusion of bacterial byproducts, inflammatory mediators, and cytokines through the pulp connective tissue might cause odontoblasts in the radicular pulp to become disordered and die, together with formation of calcifications in the pulp tissue and the atubular calcified tissue on the root canal wall.

This study provided an information that this atubular reparative dentin seemed to be a factor influencing the penetrability of root dentin. The tubular sclerosis has been proved impeding the penetration of irrigants into the root canal system [18–20]and its depositing begins in the third decade of life in the apical root region and advances coronally with age [21]. Because the patients from whom the teeth were extracted were younger than 30 years old in this study, this physiological factor was eliminated to the degree possible. To investigate the pathological change in the infected root canal, we performed histomorphometric analysis of the root dentin using a method that was developed for studying undecalcified bone specimens [25, 26]. The method used sections that had been observed for irrigants penetration, and that were ground to a thickness of 20–30 μm for histological analysis. Microscopic observation showed that more atubular reparative dentin was deposited on the walls of infected root canal.

To our knowledge, this is the first study to analyze the histopathologic features of radicular infected dentin and demonstrate an association with dentin penetrability. Previous studies have also reported that tertiary dentin formation can change the dentin permeability [27, 28]. In those studies, the authors demonstrated that under deep caries lesions and following capping of pulp exposures, the original odontoblasts were destroyed and replaced by newly differentiated odontoblast-like cells to form tertiary dentin. The tubules in the tertiary dentin not continuous or fewer in number or smaller in diameter. These factors would reduce the permeability of dentin. Consistent with previous studies, our data showed that various reparative dentin deposited on the surface of the root canal wall, which may reduce the permeability of the radicular dentin, thereby impeding the penetration of chemical irrigation into the infected dentine. However, because of our limited sample size, cautions must still be taken when extrapolating the study outcomes to a clinical setting. Additionally, it would be informative to compare the difference using a paired samples design method. To confirm this finding, more studies are needed, including the adoption of newer imaging techniques in the future.

## Conclusions

In this study, the dentin penetrability of teeth with necrotic teeth and apical periodontitis was less than that of healthy teeth. The histopathological changes of higher prevalence of reparative dentin formation in infected radicular dentin, which may influence the penetrability of infected root dentin.

## Data Availability

The datasets created and analyzed in this research are available upon reasonable request from the corresponding author.

## Abbreviations

NaOCl: sodium hypochlorite
EDTA: ethylenediaminetetraacetic acid
PUI: passive ultrasonic irrigation.
